# Multiple skin abscesses due to *Nocardia neocaledoniensis*: a case report and literature review

**DOI:** 10.1186/s12879-024-10177-7

**Published:** 2024-11-07

**Authors:** Jun Wang, Taigui Chen, Shijie Peng, Lianbao Li, Liling Wang, Jun Li, Wei He

**Affiliations:** 1 Affiliated Hospital of Panzhihua University, Panzhihua, China; 2 Panzhihua Central Hospital, Panzhihua, China

**Keywords:** *N. Neocaledoniensis*, Multiple skin abscesses, Case report, mNGS

## Abstract

*N. neocaledoniensis* is a very rare infectious pathogen that causes human disease, particularly in immunocompromised individuals. In this case report, we describe the successful diagnosis of *N. neocaledoniensis* in a patient confirmed by mNGS and the treatment of multiple skin abscesses due to *N. neocaledoniensis* infection. mNGS is an important diagnostic method complementary to routine bacterial culture and identification methods, especially for rare, novel, co-infected pathogens, and pathogens that are difficult to culture. This report may provide a reference for the clinical treatment and diagnosis of *N. neocaledoniensis* infection in humans.

## Background

The *Nocardia* species are environmental bacteria associated with human opportunistic infections, typically in immunocompromised individuals [1]. To date, there are rare reports that *Nocardia neocaledoniensis (N. neocaledoniensis)* causes skin and soft tissue infections. This bacterium has received relatively little attention in humans given the paucity of molecular diagnostics until recently [2, 3] Due to the development of diagnostic equipment to characterize microorganisms, such as matrix-assisted laser desorption ionization time-of-flight mass spectrometry (MALDI-TOF-MS), 16 S rRNA gene sequencing, and metagenomic next-generation sequencing (mNGS), more and more *Nocardia* species have been described. In this report, we present the successful diagnosis and treatment of a rare case of skin and soft tissue infection due to *N. neocaledoniensis* using mNGS.

## Case presentation

An 83-year-old man was admitted to the Department of Pneumology on November 15, 2021, due to recurrent cough and sputum with shortness of breath after activity, with back pain for more than 10 days. Admission diagnosis of interstitial lung disease, pulmonary infection, type 2 diabetes, and abdominal mass (which was suspected to have been from tuberculosis or a tumor). One month ago, the patient was hospitalized for a lung infection. During the last hospitalization, the patient’s condition had improved after symptomatic treatment with antispasmodic and asthma relief, cough and phlegm relief, hemostasis, hypotension, and anti-infection. Meanwhile, he noticed a mass (7 cm × 4 cm) on his left lower back and another (4 cm × 2 cm) on his right lower back. Both masses were firm and tender to palpation with elevated skin temperature and no redness, swelling, or ulceration. However, the patient refused further examination and was automatically discharged.

**Fig. 1:**
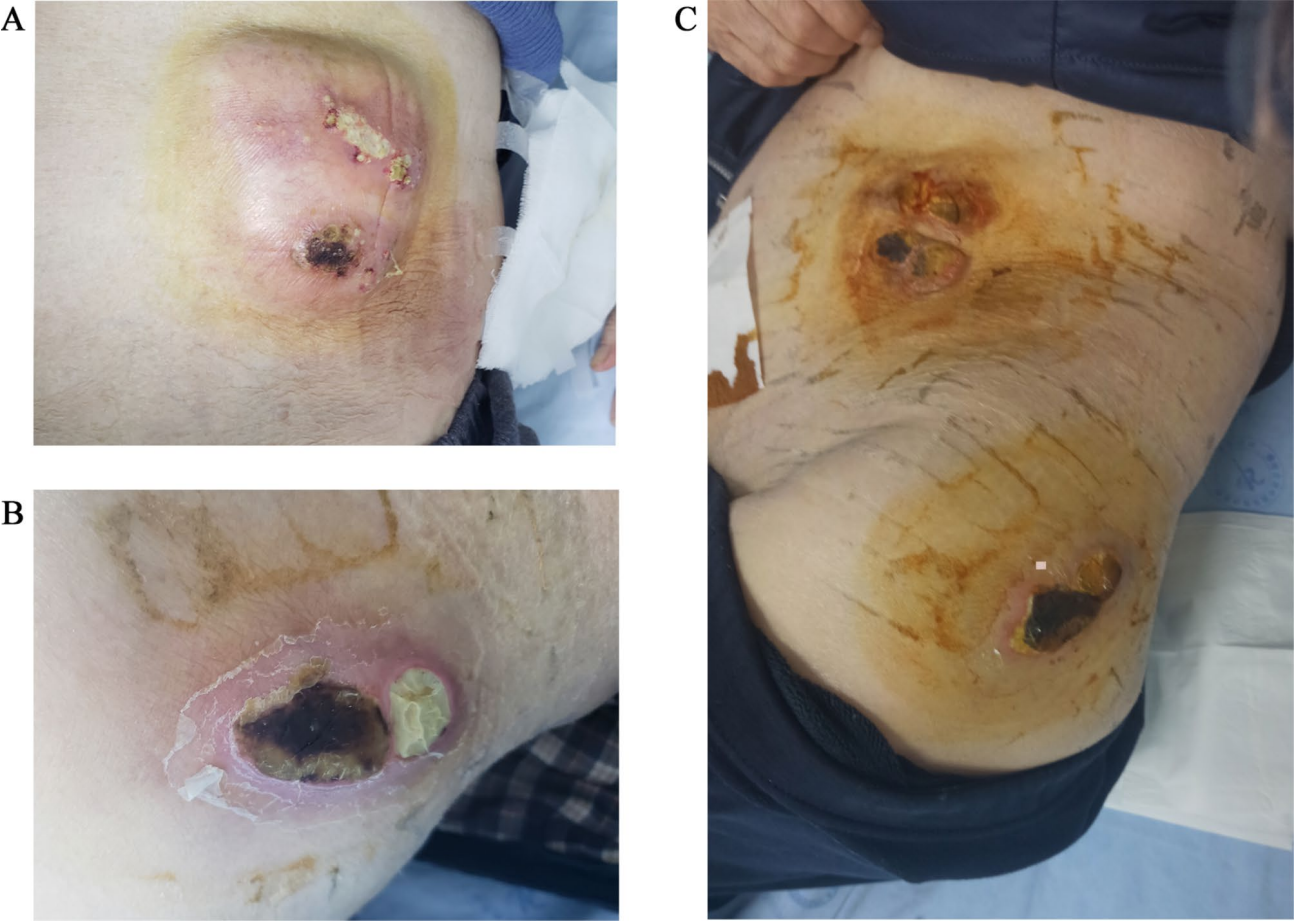
Patient’s masses (**AB** Pre-treatment; **C** Post-treatment)

**Fig. 2:**
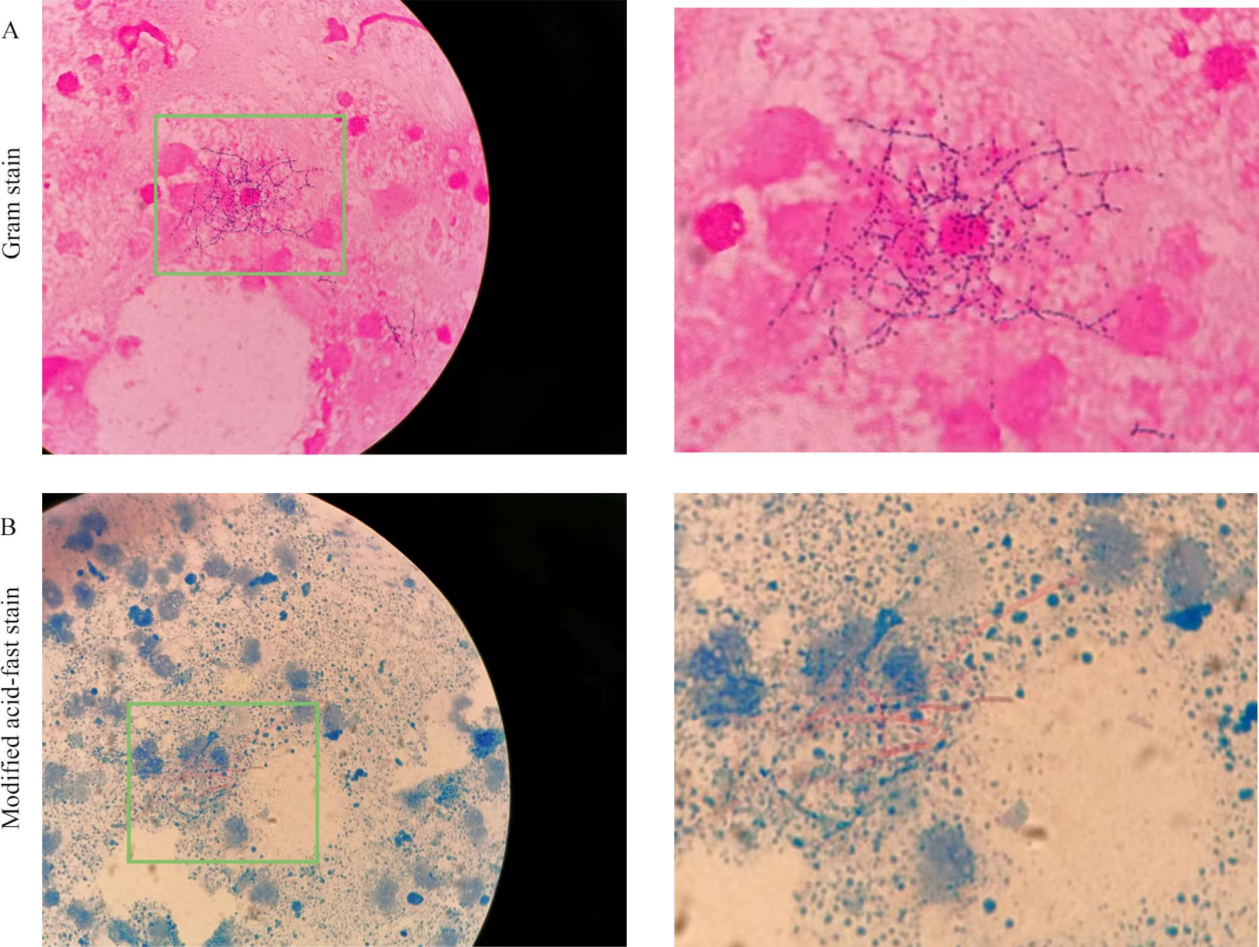
The pus sample Gram stain revealed Gram-positive bacilli (**A**) and a modified acid-fast stain-positive (**B**) at magnification 1000

**Fig. 3:**
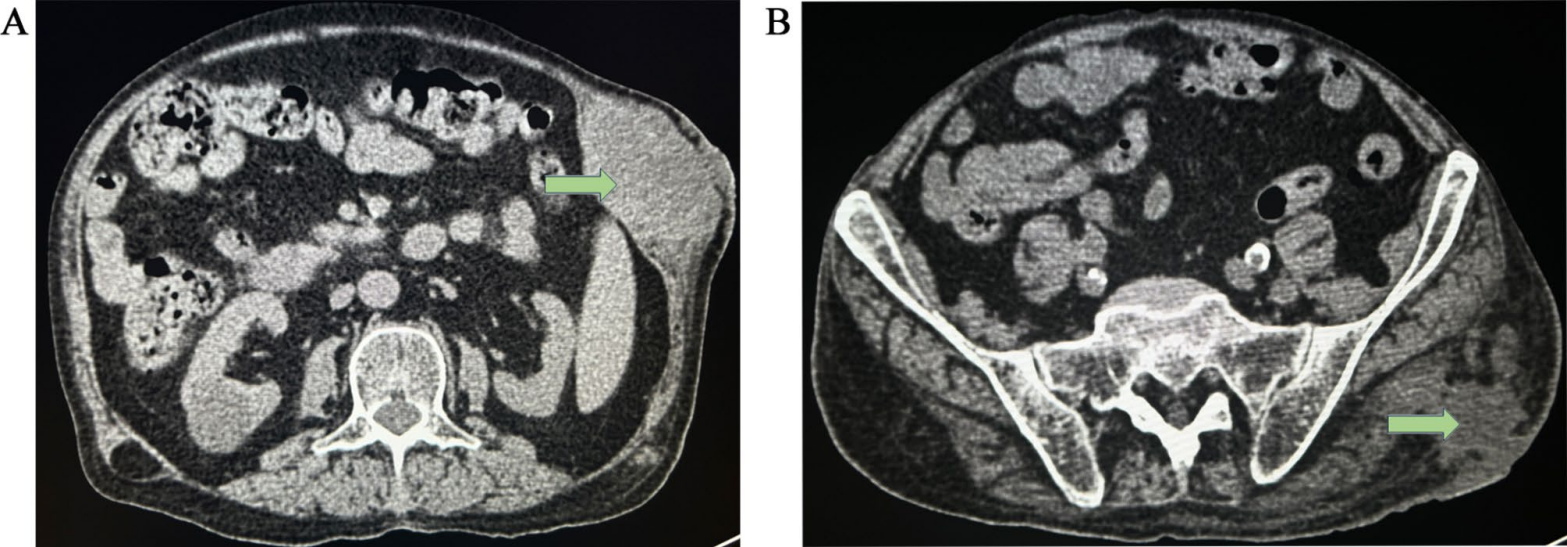
The systematic abdominal CT scan performed showed Soft tissue density mass shadow on the left abdominal wall (**A**); Subcutaneous watery density mass shadow in the left buttock area (**B**)(Arrows)

During this admission described in this report, a physical examination revealed that the patient’s right forearm had some red and swollen skin (3 cm × 4 cm), the left lower back had a mass (7 cm × 5 cm), and left hip joint had another mass (7 cm × 4 cm). Both masses were most visibly swollen or fluctuating with firm, tenderness, and redness, which had abscess spots of yellow-red pus flowing out (Fig. 1-AB). The patient’s BMI (Body Mass Index.) was 21.48. Blood examination showed the following: white blood cell count, 17.03 × 109/L; neutrophil percentage, 97.3%; C-reactive protein, 53 mg/L; and procalcitonin, 0.25 ng/ml, which illuminated an inflammatory response. The pus sample Gram stain revealed Gram-positive bacilli and a modified acid-fast stain indicative of *Nocardia* (Fig. 2). Unfortunately, the culture showed nothing on blood agar and chocolate agar media after 7 days of incubation at 35 ℃ in an atmosphere containing 5% CO_2_. On November 16, 2021, A computed tomography CT of the lower abdomen and pelvic cavity showed a soft tissue density mass shadow on the left abdominal wall and a subcutaneous watery density mass shadow in the left buttock area (Fig. 3), suggestive of an infectious lesion. Subcutaneous lipoma was considered as a possibility for the right lower back. Lumbar skin ultrasound revealed no echoes in the left lumbar region and left buttock, consider as an abscess. On November 18, 2021, a specimen was obtained by needle aspiration from the low back mass and no organisms were observed by culture and smear. Meanwhile, the specimen was detected by metagenomic next-generation sequencing (mNGS), confirming the presence of *N. neocaledoniensis.*

Upon diagnosis on November 19, 2021, the patient stopped being treated with cefazolin and ornidazole and was changed to intravenous linezolid (600 mg/q12 h) and oral trimethoprim-sulfamethoxazole (TMP-SMX) (160/800 mg, 2 tablets/6 h). On November 21, 2021, an examination revealed that the abscesses in the lower back and hip were smaller with reduced secretion. On November 25, 2021, examination revealed significantly less secretion than before. Serum infection markers, such as white blood cell count, neutrophil percentage, C-reactive protein, and procalcitonin, improved gradually (Table 1). On November 30, 2021, examination showed less low-back pain than before, and in both of the smaller masses with crusted surfaces, there was no longer any significant secretion overflow (Fig. 1-C). The patient and his family then requested discharge. He was instructed to continue oral TMP-SMX for three months, and after discharge, the patient was followed up for three months and recovered well.

**Table 1:** Biochemical parameters at critical times

Biochemical parameters	White blood cell count(×10^9^/L)	Neutrophil percentage(%)	C-reactive protein(mg/L)	Procalcitonin(ng/mL)
Pre-treatment(November 15,2021)	17.03	97.3	53.00	0.25
Post-treatmentt(November 25,2021)	5.91	89.7	6.00	0.1

Normal reference range:White blood cell count(×109/L):4.0–10.0; Neutrophil percentage(%):39.8–70.5; C-reactive protein(mg/L):0.0–5.0; Procalcitonin(ng/mL):<0.05

## Discussion and conclusions

**Fig. 4:**
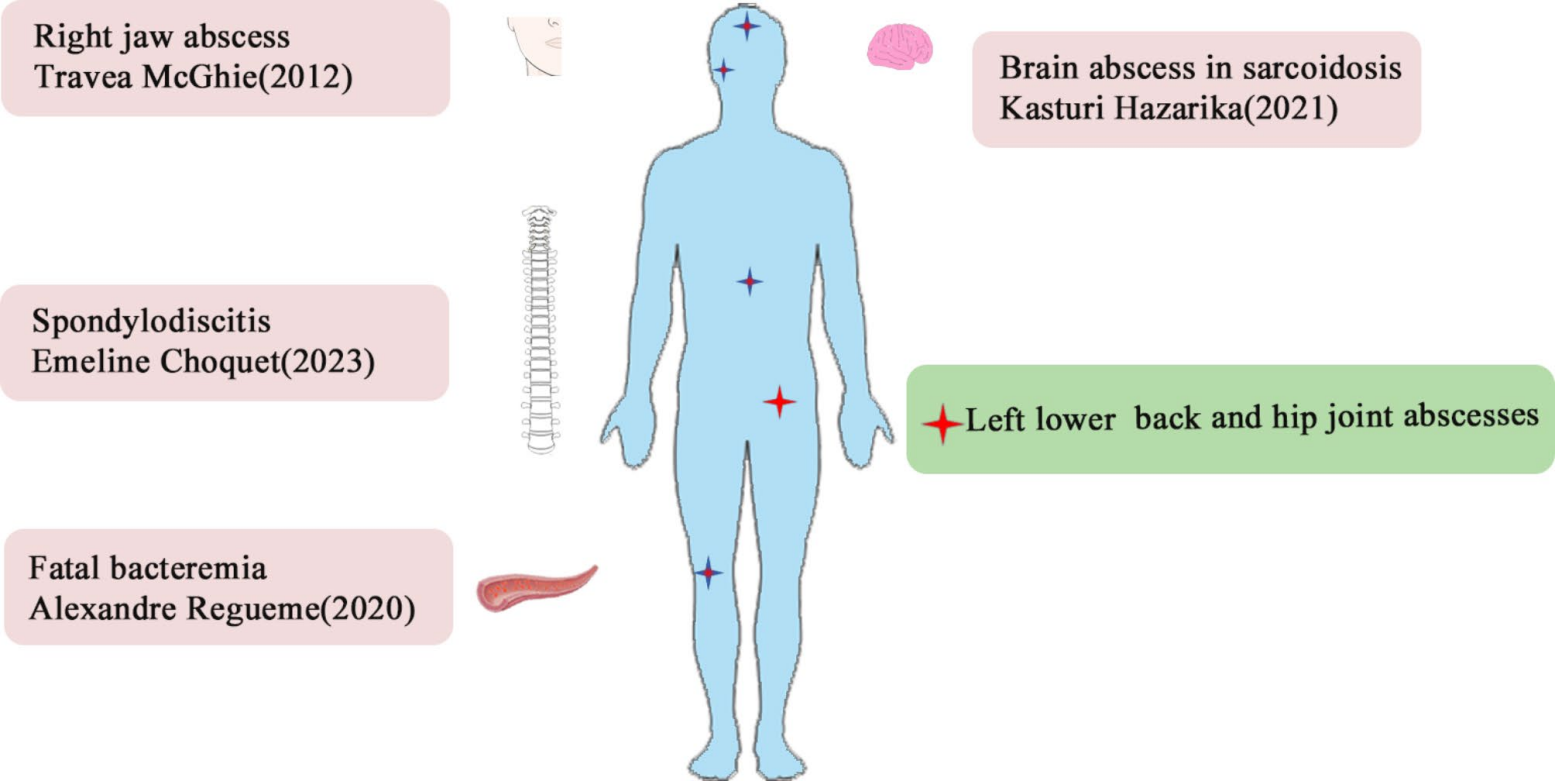
Distribution of *N. neocaledoniensis* infection cases of humans

*Nocardia* species are Gram-positive, variably acid-fast, strictly aerobic bacteria that can form branched filaments [4] and are relatively uncommon pathogens for opportunistic infections in immunocompromised patients [5]. It has been reported that the bacteria have caused lymphocutaneous, pulmonary, central nervous system, cutaneous, and subcutaneous infections [2]. The primary infection sites are the lungs and skin [3]. *N. neocaledoniensis* is a rare species of *Nocardia* bacteria, described by Saintpierre-Bonaccio for the first time in 2004 in hypermagnesian, ultramafic soil of New Caledonia [6]. In this case report, we describe the successful diagnosis of *N. neocaledoniensis* in a patient confirmed by mNGS and the treatment of multiple skin abscesses due to *N. neocaledoniensis* infection. However, few human infections of *N. neocaledoniensis* have been reported. We searched PubMed and the Web of Science for articles using the keywords “*Nocardia neocaledoniensis*.” Only 4 cases of human infection were found during the literature review (Table 2). In those cases, the bacterium was isolated by culture from spinal biopsy samples [1], blood cultures in patients with chronic lymphocytic leukemia [7], tissue and aspirate from a brain biopsy [8], and an abscess in the right jaw in a patient with rheumatoid arthritis [9] (Fig. 4). All of the patients were immunocompromised, such as with basic illness or older age. The patient in this case report was at high risk for nocardiosis because of diabetes.

**Table 2:** Review of literature of reported cases of *N. neocaledoniensis*

Case report	Gender	Age	country	Presentation	basic illness	Diagnosis approach	results of susceptibility testing	Antimicrobial	Outcome
Emeline Choquet(2023)	man	68	France	Spondylodiscitis	diabetes and rectal cancer	MALDI-TOF MS, 16S rRNA sequence ,nucleotide sequences of the secA1 (468bp) and sodA (386 bp) genes	TMP-SMX:S;linezolid:S	ceftriaxone, trimethoprim/sulfamethoxazole	recovery
Alexandre Regueme(2020)	man	88	France	Fatal bacteremia	chronic lymphocytic leukemia	MALDI-TOF MS, 16S rDNA sequence	TMP-SMX:S;linezolid:S	amoxicillin-clavulanic acid and ciprofloxacin	death
Kasturi Hazarika(2021)	woman	57	India	Brain abscess in sarcoidosis	diabetes and hypothyroidism	MALDI-TOF MS	TMP-SMX:R;linezolid:S	imipenem,amikacin ,cotrimoxazole	death
Travea McGhie(2012)	man	68	USA	Skin and Soft Tissue Infection	rheumatoid arthritis	16S rRNA sequence, amino acid sequencing of the secA1 gene	TMP-SMX:S;linezolid:S	imipenem,trimethoprim/sulfameth,doxycycline,moxifloxacin	improvement

As the first line of pathogen detection, the microbiology laboratory plays an important role in infection control by employing microscopic examination, cultures, identification, and drug sensitivity tests. The ability to quickly and accurately identify the pathogen is important for patients infected with *N. neocaledoniensis*. However, it is difficult to identify *Nocardia* isolates grown on routine culture media, which can take two weeks or longer [10]. Currently, mass spectrometry and gene sequencing are reliable and rapid identification methods. In this patient, we initially considered tuberculosis or a tumor as his diagnosis. The results of the Gram stain and modified acid-fast stain provided primary and crucial clues, and the results from mNGS revealed that the real pathogen was *N. neocaledoniensis*. Therefore, we speculate that *N. neocaledoniensis* may have been the real cause of the multiple skin abscesses. Based on this identification, the patient received timely, effective, and appropriate treatment. Unfortunately, *N. neocaledoniensis* was not able to be isolated from routine culture media in our case. Some studies report that *N. neocaledoniensis* should be isolated within 3 days of culture [1, 7–9]. We speculate that there may have been insufficient incubation time that should have been prolonged to two weeks or more.

With the development of molecular diagnostic methods [11], mNGS overcomes the limitations of other current diagnostic tests and opens another pathway for the diagnosis of infectious diseases [12] In the absence of cultured bacterial colonies and laboratory testers without basic knowledge of bacteria, it can still provide new diagnostic clues for the diagnosis of difficult pathogens in infections in critically ill or immunodeficient patients [12, 13]. Additionally, mNGS is more efficient, often requiring only 1–2 days to obtain results, which represents a significant improvement compared to traditional microbial culture and targeted antibiotic therapy could be given as early as possible [14]. This is helpful for clinicians to reduce the empirical usage of broad-spectrum antibiotics and may even affect the clinical prognosis. Meanwhile, mNGS detection technology has some drawbacks such as expensive, laborious, and complex operational procedures, contamination issues, and difficulty in distinguishing live or dead pathogens [12]. The higher detection rate of false-positive pathogens in mNGS than conventional methods. Thus, when interpreting mNGS results, it is crucial to distinguish between true-positives and false-positives of pathogenic bacteria, and this also is a new challenge for clinicians.

The susceptibility of various pathogenic *Nocardia* to antimicrobial agents varies considerably. Therefore, identification at the strain level is crucial to using effective antibiotics for appropriate empirical treatment in clinical practice [15]. Some reports show that trimethoprim-sulfamethoxazole (TMP-SMX) and linezolid are effective against most *Nocardia* species [15]. As a result, TMP-SMX is recommended as the initial drug of choice [2, 15, 16]. However, long-term antibiotic exposure increases more opportunity for the evolution of resistance [3]. Especially in immunosuppressed patients, cases of therapy failure due to *Nocardia* resistance are increasingly reported [17, 18]. A recent study suggests that *N. neocaledoniensis* isolated from tissue and aspirate from a brain biopsy was resistant to TMP-SMX [8]. The patient in our report was fortunate that the empirical combination therapy of linezolid and TMP-SMX was effective despite lacking the results of susceptibility testing. Inevitably, due to the lack of susceptibility testing and result of antibiotic resistance, clinicians often prescribe antibiotics empirically to patients, which can exacerbate reinfection and drive the emergence of antibiotic resistance and multidrug-resistant pathogens [19]. Therefore, in the context of emerging diagnostic technologies, when we lack susceptibility testing and result of antibiotic resistance, mNGS can provide resistance gene information for the prediction of antibiotic resistance, and the presence of virulence factors [20].

In conclusion, this is the first report of human multiple skins and soft tissue abscesses due to *N. neocaledoniensis* as detected by mNGS. It provides another case of this species as a human pathogen. So far, molecular biology-based methods are considered to be the best option for accurate identification [1]. Moreover, mNGS has more advantages in detecting pathogens and predicting resistance. With the rapid development of sequencing technology, mNGS will soon become a routine technique in clinical laboratories and may play an important role in the treatment of human infections.

## Data Availability

The datasets used and/or analyzed during the current study are available from the corresponding author upon reasonable request.
